# Evolution of the chicken Toll-like receptor gene family: A story of gene gain and gene loss

**DOI:** 10.1186/1471-2164-9-62

**Published:** 2008-02-01

**Authors:** Nicholas D Temperley, Sofia Berlin, Ian R Paton, Darren K Griffin, David W Burt

**Affiliations:** 1 Department of Genomics and Genetics, Roslin Institute (Edinburgh), Roslin, Midlothian EH25 9PS, UK; 2 Department of Evolutionary Biology, Evolutionary Biology Centre, Uppsala University, Norbyvagen 18D, 752 36 Uppsala, Sweden; 3 Department of Biosciences, University of Kent, Canterbury, CT2 7NJ, UK

## Abstract

**Background:**

Toll-like receptors (TLRs) perform a vital role in disease resistance through their recognition of pathogen associated molecular patterns (PAMPs). Recent advances in genomics allow comparison of TLR genes within and between many species. This study takes advantage of the recently sequenced chicken genome to determine the complete chicken TLR repertoire and place it in context of vertebrate genomic evolution.

**Results:**

The chicken TLR repertoire consists of ten genes. Phylogenetic analyses show that six of these genes have orthologs in mammals and fish, while one is only shared by fish and three appear to be unique to birds. Furthermore the phylogeny shows that TLR1-like genes arose independently in fish, birds and mammals from an ancestral gene also shared by TLR6 and TLR10. All other TLRs were already present prior to the divergence of major vertebrate lineages 550 Mya (million years ago) and have since been lost in certain lineages. Phylogenetic analysis shows the absence of TLRs 8 and 9 in chicken to be the result of gene loss. The notable exception to the tendency of gene loss in TLR evolution is found in chicken TLRs 1 and 2, each of which underwent gene duplication about 147 and 65 Mya, respectively.

**Conclusion:**

Comparative phylogenetic analysis of vertebrate TLR genes provides insight into their patterns and processes of gene evolution, with examples of both gene gain and gene loss. In addition, these comparisons clarify the nomenclature of TLR genes in vertebrates.

## Background

Toll-like receptors (TLRs) perform a vital role as sentinels of the innate immune system in their host organism through the recognition of pathogen associated molecular patterns (PAMPs). PAMP recognition is achieved through binding to the extracellular leucine rich repeat (LRR) containing domain, specific to each receptor type [1]. In response to ligand binding, signalling is activated by the cytoplasmic Toll/interleukin I resistance (TIR) domain resulting in an inflammatory response and release of inflammatory cytokines. This is an evolutionarily highly conserved domain, present in a wide range of taxa from plants to animals indicating the first Toll-like gene existed in the unicellular ancestor of plants and animals over 1 billion years ago [2-6,1].

The first Toll gene to be discovered was in *Drosophila melanogaster*, which encodes a receptor involved in developmental patterning, but more importantly also in resistance to fungal infections [7]. In animals, these genes can be sub-divided into two simple categories based on taxonomy; the Toll genes of Protostome taxa and the TLRs of Deuterostomes [5,6]. It is likely that Toll originally had a purely developmental role and that this during the course of evolution became combined with disease resistance functions in coelomate organisms, to protect the body cavity from infectious pathogens [3,5].

Although TLRs have been identified in many animal lineages the majority of research has been carried out on eutherian mammals, especially humans and mice [8,9]. In recent years, however, advances in teleost genomics have led to the rapid discovery of TLR genes in fugu and zebrafish [10-12]. Moreover, recent analyses of chicken ESTs and genome sequences have revealed TLRs in chicken [13-17].

Gene gain and gene loss are important evolutionary processes, especially with respect to gene family dynamics [18-22]. Gene duplication is believed to be the principal cause by which new genes are created. Gene gain can occur either on a large scale from a whole genome duplication or on a small scale when chromosomal sections or individual genes are copied. Single gene duplications occur by tandem duplications as a result of either unequal crossing over or by retrotransposition [19]. Retrogenes are clearly recognised by their lack of introns and are often found to be non-functional paralogs, as regulatory sequences are usually missing [23]. Gene gain is frequently followed by differential gene loss either by mutation in one gene isoform leading to the creation of a pseudogene or by complete deletion [24-26]. It is believed that gene deletion closely follows duplication in the majority of cases [27]. The process of gene gain and gene loss is believed to occur at a constant rate with the exception of an episode of whole genome duplication in vertebrates ~500 Mya [22]. On occasions where both genes remain active one may be freed from purifying selection and be able to evolve a new function, or it may retain its original function but alter its tissue expression profile [18,28,26].

To define TLR orthologs and uncover the pattern of gene duplication and/or gene loss in vertebrate lineages, we performed a phylogenetic analysis of all known TLR sequences. Our results confirm all of the chicken TLR genes found in previous studies [13-17] and do not detect any more. Our findings also confirm and extend the sequence and structure of these genes and corroborate the presence of duplicated genes as being separate genes and not splice variants of the same gene.

### Aims

The major aims of this investigation were to determine what TLRs are present in chicken and determine where unmapped TLR genes are located. These data then allowed us to define orthologs in other species and examine TLR gene family evolution using phylogenetic methods. These analyses allowed us to characterise the variation within and between vertebrate lineages, as well as the dynamics of TLR gene gain/gene loss. In addition, TLR gene nomenclature issues were also addressed.

## Results

### Definition of orthologs and TLR gene nomenclature

Sequences newly identified as TLRs and those that have previously been named are listed in Table 1. Suggested alterations to nomenclature are devised in accordance with the standards of human genome nomenclature. Of particular note is the sequence Genbank: AY531552, which has sometimes previously been described as mouse TLR11 [29,30] although it shares its sequence with Genbank: AK136724, AK143385 and AY510705, all of which are described as TLR12. Phylogenetic and structural analyses combined with gene mapping data in mouse and rat have led to this investigation regarding AY531552 as mouse TLR12. Our phylogenetic analyses showed the *Lethenteron japonicum *(lamprey) TLRs to be duplicated forms of TLR14 and various fish TLRs to be TLR22. A TLR5 in *Xenopus laevis *was also defined [Genbank: BC084773].

**Table 1:** Suggested changes in nomenclature of TLR genes

**Organism**	**Name we propose**	**Previous name**	**Accession**
Chicken	TLR15	TLR 2 variant 2	XM_419294
	TLR21	hypothetical protein LOC415623	NP_001025729
	TLR1LA	TLR1 type1, TLR1/6/10, TLR1, TLR16	AB109401
	TLR1LB	TLR1 type2, TLR2, TLR6	DQ518918
	TLR2A	TLR2, TLR2 type1	NM_204278
	TLR2B	TLR2 type2	AB046533
*Xenopus laevis*	TLR5	hypothetical LOC495313	BC084773
Lamprey	TLR14a	TLRa	AB109402
	TLR14b	TLRb	AB109403
Rat	TLR13	similar to toll-like receptor	XM_228540
Japanese flounder	TLR22	TLR3	AB109396
Goldfish	TLR22	TLR	AY162178
Rainbow trout	TLR22a	TLR	AJ628348
	TLR22b	TLRII	AJ878915
mouse	TLR12	TLR11	AY531552

Current name indicates those used by this paper following detailed phylogenetic and structural analyses.

### TLR gene discovery in chicken and other vertebrates

We used a total of 143 non-redundant TLR sequences (Additional file 1) in this study, representing 26 TLR genes from 30 different species as described in Table 2.

**Table 2:** Function and taxonomic presence of known TLRs

**Name we propose**	**Known host taxa**	**Ligand**	**Pathogen**	**Example accession**	**References**
TLR1	Teleostei	Unknown	Mycobacteria	AC56430	[10]
TLR1LikeA	Aves	Unknown	Unknown	AB109401	[17]
TLR1LikeB	Aves	Unknown	Unknown	DQ518918	[17]
TLR1	Eutheria	Lipopeptide	Bacteria	AY009154	[3]
TLR2	Vertebrata	Lipopeptide and peptidoglycan	G+ bacteria	AC156432, NM_204278, NM_011905	[3]
TLR3	Vertebrata	dsRNA	Viruses	AC156436, NM_001011698, AF355152	[3]
TLR4	Vertebrata, but lost in most teleosts	LPS	G-bacteria	AY388400, AY064697, BC029856	[3, 10]
TLR5	Vertebrata	Flagellin	G-bacteria	AC156437, AJ626848, AF186107	[3]
TLR5S	Teleostei	Flagellin	Bacteria	AB062504	[36]
TLR6	Eutheria	Lipopeptide and Zymosam	Bacteria	BC055366	[3]
TLR7	Vertebrata	Imiquimod	Viruses	AC156438, NM_001011688, AY035889	[3]
TLR8	Teleostei and mammalia	Imiquimod	Viruses	AC15639, AY035890	[3]
TLR9	Teleostei and mammalia	CpG motifs	Bacteria and viruses	AC156432, AF314224	[3]
TLR10	Mammalia	Unknown	Unknown	XM_223422	[3]
TLR11	murinae	Unknown	Unknown	AY501704, XM_373751	
TLR12	Mammalia	Profilin	Uropathogenic bacteria	AY351552*, AY510705, XM_342922	[29, 30]
TLR13	Amphibia and Mammalia	Unknown	Unknown	AY510706	
TLR14	Amphibia, Teleostei and Hyperoartia	Unknown	Unknown	AC156413	[15]
TLR15	Aves	Unknown	Unknown	XM_19294	[13]
TLR16	Amphibia	Unknown	Unknown		[15]
TLR18	Teleostei	Unknown	Mycobacteria	XM_682223	[10]
TLR19	Teleostei	Unknown	Unknown	XM_68516	
TLR20	Teleostei	Unknown	Mycobacteria	no full length sequence available	[10]
TLR21	Teleostei and Aves	Unknown	Unknown	AB101002, NM_001030558	[10, 15]
TLR22	Teleostei	Unknown	Mycobacteria	AC156434	[10]
TLR23	Teleostei	Unknown	Unknown	AC1564345	

TLRs are listed in numerical order along with pseudonyms found by this investigation and example GenBank accession numbers. Taxonomic coverage is inferred by the presence of sequences found in this investigation and corresponding phylogenetic data. The PAMP and pathogen data are reported as found in the cited literature.

* incorrectly described as TLR11 in the literature

This investigation confirms the presence of the 10 TLRs in the chicken found in previous studies [13-17] and found no further TLRs. This is the same number as found in human and two fewer than in mouse. Chicken TLRs 3, 4, 5 and 7 are directly orthologus to those found in other vertebrates. The duplicated genes, TLRs 2A and 2B found in the chicken are both orthologs of the single TLR2 of mammals. Chicken TLR21 is an ortholog of TLR21 in fish and amphibians. It appears that TLRs 1LA, 1LB and 15 are unique to birds (see Tables 2 and 3).

**Table 3:** Chicken TLR details

**Given name**	**Chromosome**	**Location**	**Exons**	**Length (aa)**	**Length (genomic)**	**Length (CDS)**	**Described**	**Genbank nucleotide**	**Genbank protein**
TLR1LA	4	71563594–71566050	1	818	2457	2457	[15]	AB109401	BAD67422
TLR1LB	4	71553122–71555080	1	722	5005	1959	[15]	DQ518918	ABF67957
TLR2A	4	21105675–21108056	1	793	2382	2382	[13, 31]	NM_204278	NP_989609
TLR2B	4	21113342–21115936	1	781	2594	2346	[13, 31]	AB046533	Q9DGB6
TLR3	4	63155888–63160902	4	896	5015	2691	[13-15]	NM_001011691	NP_001011691
TLR4	17	4062994–4067445	3	843	4452	2532	[13-15]	AY064697	AAL49971
TLR5	3	18975945–18978530	1	862	2589	2589	[13, 14]	AJ626848	CAF25167
TLR7	1	126824071–126830542	2	1059	6381	3180	[13-15]	NM_001011688	NP_001011688
TLR15	3	2945856–2948462	1	868	2607	2607	[13]	XM_419294	XP_419294
TLR21	11	338885–342202	2	972	3317	2919	[15]	NM_001030558	NP_001025729

The chicken TLR repertoire contains tandem duplications of the genes for TLRs 1L and 2 [31,17]. A partial sequence of chicken TLR1LB has been described previously by Yilmaz et al. [17]. Using RT-PCR we have sequenced and cloned the full length mRNA [Genbank: DQ518918]. This sequence includes two further exons which, although transcribed, are not translated. Using synonymous substitution rates (see Materials and Methods) we estimated the gene duplication events that gave rise to TLR1LA, 1LB and 2A, 2B to have taken place 147 and 66 Mya respectively. The chicken is also found to be missing a number of TLRs, which are present in most mammals. The TLR7, 8 and 9 subfamily is present in fish and mammals but is only represented by TLR7 in the chicken [14,16,17]. TLR8 is present as a pseudogene [32] and TLR9 has been deleted.

### Genetic Mapping of chicken TLR1LA and B genes

The chromosomal locations of most chicken TLRs are known [17], with the exception of TLR1LA and TLR1LB which, based on a single sequence contig [Genbank: NW_001471687] map close together on an unmapped chromosome [17]. Sequencing of a genomic fragment from TLR1LB locus from parents of the East Lansing reference mapping cross, identified two SNPs in TLR1LB. Genetic linkage analysis of TLR1LB showed linkage to chromosome 4 (see Table 3).

### Structural analysis of proteins and selective constraints on functional domains

**Figure 1:**
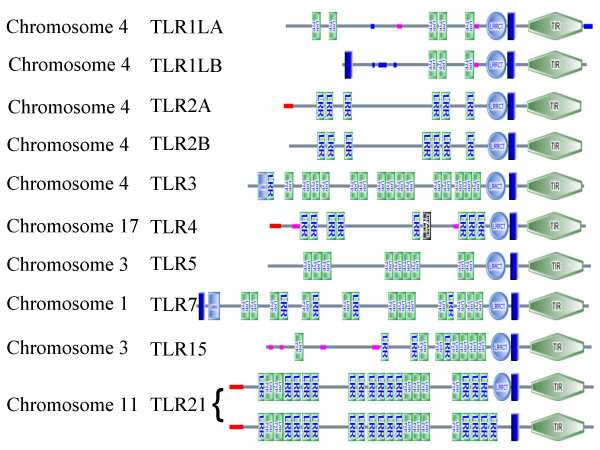
**Chicken TLR structures**. SMART structure of chicken TLRs. Structures joined by { shows the two equally likely structures of TLR21. Dark blue rectangles represent trans-membrane regions while green bands indicate LRRs involved in PAMP recognition (LRR-TYP represents typical LRRs, whereas LRR represents LRR outliers). Light blue rectangles and circles represent L terminal and C terminal LRRs respectively, thin red bars represent signal peptides and thin pink bars segments of low compositional complexity. Motifs with the prefix PFAM represent those that are recognised by the PFAM database.

Comparisons of structural predictions for the chicken TLRs show them to have a varied structure [17]. The newly discovered TLR15 has the unusual feature of many LRRs clustered towards the C-terminus of the molecule and few at the N-terminus (Figure 1). TLR7 has the uncharacteristic feature of a splice variant with two predicted trans-membrane (TM) domains only previously found in mammalian TLR6 (see Additional file 2). The predicted structure of the TLR1LB fragment described by Yilmaz et al. [17] is shown to have a further predicted TM domain towards the N-terminus whereas the majority of TLRs have a single TM domain between the extracellular and intracellular domains (Additional File 2). The predicted structure of this TLR is otherwise very similar to TLR1LA only missing the two LRRs at the N-terminus (see Figure 1). By contrast the duplications of TLR2 are marked by the absence of an LRR from TLR2A, which is present in all other known TLR2s (Additional File 2).

### Phylogeny of vertebrate TLRs

**Figure 2:**
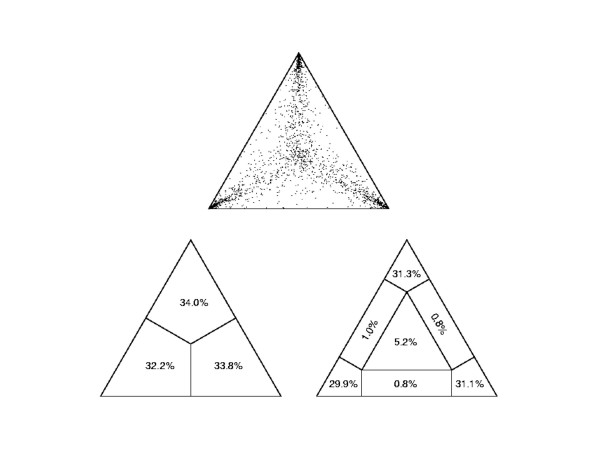
**Likelihood map of TLR protein alignment**. The high density of points in the apexes of the triangle indicates the strong amount of structure and therefore great suitability for phylogenetic analysis.

**Figure 3:**
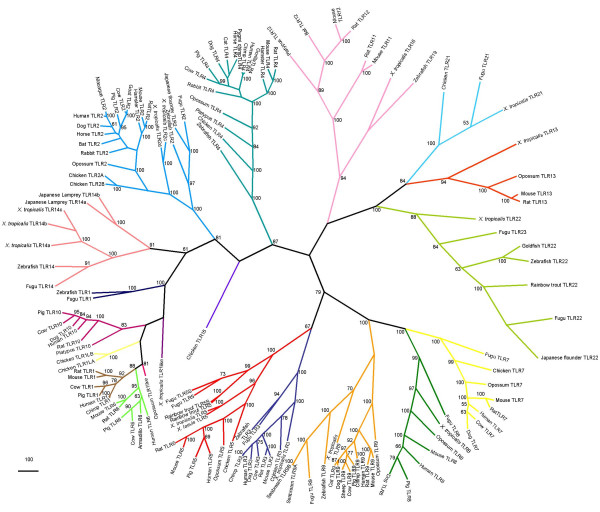
**Phylogeny of TLR protein data using the Neighbour Joining method**. Phylogeny is unrooted. The numbers at the nodes indicate percentage bootstrap values of the 1000 bootstrap replicates, only values greater than 50 are shown.

**Figure 4:**
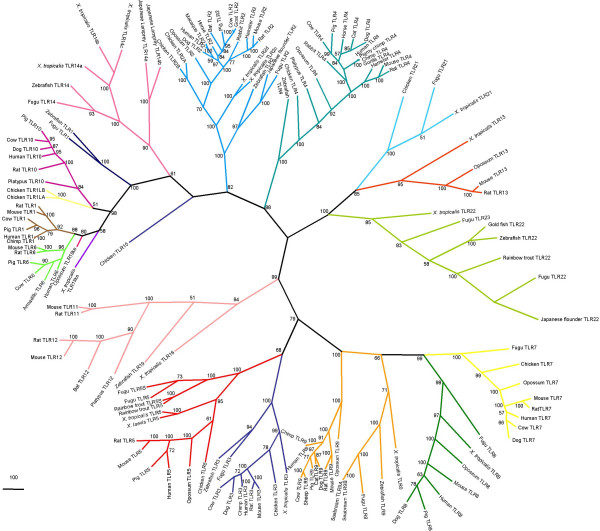
**Phylogeny of TLR protein data using Maximum Parsimony**. Of 1386805 rearrangements tried three equally parsimonious trees were found, phylogeny is unrooted. The numbers at the nodes indicate percentage bootstrap values of the 1000 bootstrap replicates, only values greater than 50 are shown.

Molecular phylogenies based on sequence alignments are only as accurate as the alignment data from which they are produced; consequently it is important that the alignment data quality is determined [33-35]. In order to assess the alignment's tree like structure, likelihood mapping was carried out and showed the sequence alignment data to have strong phylogenetic signal (Figure 2). Both phylogenetic methods used produced near identical topologies, which were strongly supported with bootstrapping (see Figures 3 and 4). Close up images of individual clades in Fig. 3 are shown in Additional files 4, 5 and 6. Close up images of individual clades in Fig. 4 are shown in Additional files 7, 8 and 9.

The majority of TLRs form specific clades, with each species containing one copy of each receptor (e.g. TLRs 3, 4 and 5). The main exception to this trend is the assemblage of receptors named TLR1, which have independently evolved in fish, *Xenopus*, chicken, opossum and eutherian mammals (see Figures 3 and 4). These genes are not orthologs but have often been given the same name, which creates some confusion (see Table 1). The same is true for TLR5S in fish, where a soluble TLR5 has independently evolved in different fish species [36] (see Figures 3 and 4). The presence of most TLR gene families across so great a range of vertebrate taxa indicates that the majority of TLR families must have been established prior to the evolutionary radiation of vertebrates in the Cambrian Period 540-505 Mya [37].

Phylogenetic analyses show that chicken TLR21 is an ortholog of fugu and *Xenopus *TLR21. Chicken TLR15 shares a clade with TLRs 1, 2, 6, 10 and 14 (Figures 3 and 4), but the precise nature of this relationship remains unresolved due to the small number sequences available. TLRs 3, 5, 7, 8 and 9 form a clade that mainly follow the expected species tree; the exceptions to this being the loss of chicken TLRs 8 and 9 and the gain of soluble versions of TLR5 in teleost fish. Gene gain has been comparatively common in the clade consisting of TLRs 2, 14 and the 1, 6, 10 assemblage. The TLRs in this clade are found to respond to bacterial pathogens, and in mammals and chicken have been shown to function heterodimerically [3,38].

No specific ligand has been identified for the clade containing TLR 13, 21 and 22 (Table 2). The neighbour joining tree (Figure 3) attaches the TLR 11–12 clade to this group. The positioning of this clade is not as well supported by neighbour joining as it is using maximum parsimony, which places the TLR11–12 clade basal to the TLR3, 5, 7, 8 and 9 group (Figure 4). Both the TLR11–12 clade and TLR13-21-22 clade share a history of multiple gene loss events. TLRs 14, 22 and 23 have been found to have been lost from all land animals (see Figures 3 and 4).

## Discussion

Structural and phylogenetic analyses suggest the chicken TLR repertoire to consist of ten genes. Phylogenetic analyses show these to consist of six orthologs of mammals and fish, one fish ortholog and three unique to chicken. Chromosome mapping of chicken TLR genes shows them to be in syntenic positions compared to other animals. Half of the chicken TLR genes are located on chromosome 4 (Table 3). This large proportion of TLR genes on a single chromosome is due in part to the gene duplication TLRs 1L and 2 in the chicken (Figures 3 and 4). The homologues of these TLR genes in the human also map to a single chromosome [17] although this tendency is not found in other vertebrates.

### Comparison of the predicted structure of Toll-like receptors

From protein alignments as well as SMART predictions it is evident that the TIR domain is highly conserved across many different receptor families whereas the extracellular LRR region shows great variation in the positioning of LRRs between receptors although a strong level of structural conservation across taxa for a given receptor (see Additional File 2).

Chicken TLR7 has a predicted TM domain at the C-terminus as have the majority of TLRs, but also has an extra TM domain at the N-terminus. Similarly an extra TM domain at the N-terminus tip is predicted for Chicken TLR1LB as well as TLR6, a receptor present only in eutherian mammals. A TM domain is predicted at the N-terminal in fugu TLR1 but in this case the usual TM domain is not found at the C-terminal (Additional File 2). As the predicted N-terminal TM domain of TLR 1LB is also found in a number of related TLRs it is possible that this is a conserved feature. However, the absence of such a predicted structure from all examples of TLR7 other than chicken may mean that this feature is a result of the SMART program falsely interpreting a hydrophobic region. A pseudogene caused by an insertion event for TLR8 in chicken has been identified but TLR9 in chicken has been lost leaving only flanking regions where the TLR9 locus is predicted [32].

### Phylogenetic analyses of the Toll-like receptor gene family

There have been previous phylogenetic analyses of TLR data [4,10,15,17]. Since the inclusion of more sequences improves the data quality of the alignment and consequently the phylogenies produced [19,39], this investigation has been able to improve on phylogenetic analyses carried out by previous studies owing to the increased number of sequences available. This is corroborated by the likelihood mapping analysis (Figure 2) and the high bootstrap values by both neighbour joining (Figure 3) and parsimony (Figure 4) methods for the major TLR family groups.

Also very similar trees are produced from neighbour joining and maximum parsimony analyses (Figures 3 and 4).

### Gain of Toll-like receptor genes

The vast majority of duplications of whole TLR genes occurring since the Cambrian period have occurred in the clade containing TLRs 1, 2, 6 and 10. The first of these took place 300 Mya leading to the establishment of TLR 10 in the lineage that gave rise to the mammals. This was followed by TLRs 1 and 6 in the eutherian mammals 130 Mya [4]; data which are supported by Figures 3, 4 and Roach et al. [15]. A further duplication has occurred 147 Mya in the lineage that gave rise to modern birds, leading to avian TLR1LA and TLR1LB followed by the latest duplication 65 Mya producing TLR2A and TLR2B in the chicken. The duplication of TLR2 in the chicken lineage is not a unique event, other independent duplication events of TLR2 have occurred in *X. tropicalis *(Figures 3 and 4), the American alligator [4] and the ancestor of marsupial and eutherian mammals [15] the timing of these duplications, however, remains unknown.

All of the LRRs of TLR1LA are in positions shared by closely related TLRs (Additional File 2). The much shorter TLR1LB has an almost identical 3' sequence and structure, but the two 5' exons and the start of the third and final exon are disrupted by stop codons and frame shifts. A start codon is found a short way into the third exon producing a product lacking the two N-terminal LRRs and including an extra predicted TM domain. The predicted structure of this receptor is the same as that reported in Yilmaz et al. [17] although the complete coding sequence of this gene is now complete (this paper) [GenBank: DQ518918].

### Loss of Toll-like receptor genes

Compared to the paucity of gene gain in the evolution of the TLR family there are many instances of gene loss. TLRs 14, 22 and 23 are not found in any land dwelling vertebrates and TLR4 has been lost in the majority of studied fish species, yet is preserved in land animals. TLRs 11, 12 and 13 found in a small number of animals are lost in birds and many mammals, however a pseudogene for human TLR12 has been reported [30]. Although TLR19 of the zebrafish and TLR16 of *X. tropicalis *clearly represent ancestral sequences of TLRs 11 and 12 (see Figures 3 and 4), there is no trace of a receptor of this kind to be found either in other fish, mammals or the chicken. This indicates that members of this group must have been lost on more that one occasion. Functional genes for TLRs 8 and 9 are absent from the chicken; TLR8 is present as a pseudogene [32] and TLR9 has been deleted from the genome.

The inclusion of certain taxonomic groups would assist in our understanding of these gene losses. The inclusion of fish species (other than the ray-finned fish) and more basal tetrapods (squamata, anapsida etc.) would help to give a more precise idea of the point at which TLRs 22, 14 and 19 were lost.

## Conclusion

We found chicken to have a total of ten TLRs, the same number as in humans but with only five human orthologs. Phylogenetic analyses show TLR families to be of ancient origin, predating the divergence of major vertebrate lineages. The chicken genome highlights recent instances of both gene loss and gene gain within TLRs which allows an insight into the patterns and processes of gene family evolution.

## Methods

### Sequence databases

Candidate chicken TLR genes were identified using extensive searches for sequence homology using BLAST started by using the mouse and human sequences identified in Smith et al. [16] and the TIR protein consensus sequence from Meijer et al. [10] in the NCBI database [40] and the Ensembl genome browser [41]. Full coding region nucleotide and corresponding protein sequences were collected for as great a taxonomic range as possible as this improves the validity of the phylogenetic analysis [39]. Collected sequences were used in BLAST searches both into specific genomes and the general sequence database with the intention of identifying all available TLR sequences.

The full sequence for chicken TLR1LB was determined using the original version of partial sequence Genbank: AY633573, the contig Genbank: NW_001471687 (the details described in Yilmaz et al. [17]) and EST Genbank: CD762233. The complete sequence was confirmed by sequencing cDNA fragments produced by RT-PCR (for list of primers see Additional File 3). To ensure that all chicken TLRs had been identified, Hidden Markov models (HMMs) were used to analyse the chicken genome [42]. When expected TLR homologues were found to be absent from the chicken (e.g. TLR9), the sequence contig for the expected chromosomal region was identified to ensure that the absence of a TLR was not the result of a gap in the genome sequence.

### Bioinformatics

Complete protein sequences were assembled and labelled in BioEdit [43] before being aligned using ClustalX [44]. This program automatically produces a phylogeny that can be viewed in TreeView [45], which can be used as a rough guide although it is not suitable for in depth phylogenetic investigation [33]. The alignment was checked by visual inspection. To ensure that the alignment dataset was suitable for phylogenetic analyses likelihood mapping was carried out using the program TreePuzzle [46]. This method, although little used, is of great value for confirming the suitability of a dataset for use in tree building [35]. Likelihood mapping works by breaking down the alignment into quartets of sequences and assessing the likelihood of their three possible phylogenetic topologies. This information is displayed as points in an equilateral triangle where points located in the apexes represent strong 'tree like' phylogenetic signal, points between apexes give equal likelihood for two different tree topologies and points in the centre indicating 'star like' evolution [35,46,47].

A convenient way to infer events of gene duplication and gene loss is to note to what extent the gene phylogenies match the expected species phylogeny [20,21,48]. Incongruence in the close association between the two can usually be explained by episodes of gene duplication and/or gene loss in a given lineage [18,20,21,34,48]. Gene duplication followed by positive natural selection is seen as the principle means by which new gene functions arise [26,49]. A study relating to the evolution of gene families on the chordates found the majority of duplications to have occurred early in chordate evolution. This can be easily maintained by the similarity between the orthologous genes in different species being greater than that found between members of the same gene family in the same species [50,51].

Phylogenies were created in PAUP [52] using both parsimony and neighbour-joining methods. These two methods are used as each one analyses the data in different ways [19,33]. Consequently, if tree topology is not shared by both methods it shows that these relationships may not represent evolutionary heritage. To determine the support for individual clades, 1000 bootstrap replicates were carried out on the trees produced by each method [53].

The structure of chicken TLRs was predicted using the SMART program [54] to analyse full length amino acid sequences.

### RNA extraction and tissue sources

Tissues were collected and stored at -80°C from a *Gallus gallus *broiler presumed to be healthy. All tissues were ground under liquid nitrogen with a pestle and mortar. 100 mg samples of each tissue were trizol extracted and RNA quantified using a bench top spectrophotometer. To ensure RNA extractions were free from genomic DNA contamination, RNase free DNase (Promega) was used in accordance with the manufacturer's instructions. RNA samples were reverse transcribed using SuperScript II reverse transcriptase (Invitrogen Carlsbad CA) following the manufacturers guidelines, including the use of RNase H to remove complimentary RNA from the newly synthesised cDNA. All cDNAs were standardised using RT-PCR against the reference gene, GAPDH and the absence of contamination determined by the absence of bands in samples that had not undergone reverse transcription.

### Reverse-transctriptase PCR (RT-PCR) and PCR

PCR was carried out in 10 μl volumes consisting of 10 pg template, 10× dNTPs (2 mM each), 10× PCR buffer, 5× GC solution, 0.4 U Taq and 5 pM each primer. List of primer sets used is shown in Additional file 3. PCR conditions for all reactions were as follows: 15 min 95°C followed by 30 cycles of 95°C for 30 seconds, 60°C for 30 seconds and 72°C for one minute followed by an extension step of 72°C for 5 minutes. Amplified PCR products were run by electrophoresis in a 1% agarose gel stained with ethidium bromide and visualised using an ultra violet transilluminator.

### DNA sequencing

As the sequence of TLR1LB was unclear from sequence files and EST data, the full sequence was confirmed using primers shown in Additional File 3. To improve sequence quality of TLR1LB TOP sequence (see Additional File 3) we collected PCR products from agarose gels using the QIAEXII gel extraction kit (Qiagen Hilden). Inserts were grown in bacterial colonies on agar plates before colonies were inoculated into LB medium and plasmids purified using the QIAprep Miniprep kit (Qiagen Hilden). For all other sequences (those used in the SNP analysis) PCR products were purified using ExoSap and sequenced in both directions using the BigDye^® ^Terminator v3.1 Cycle sequencing kit.

### Genetic linkage mapping of Toll-like receptor genes

Two single nucleotide polymorphisms (SNPs) were identified in the TLR1LB expression fragment (for primer list see Additional File 3) from Red Jungle Fowl and White Leghorn parent birds [55]. Sequences from offspring were sequenced and genotyped and these data used for genetic mapping. TLR1LA is known to map closely to TLR1LB [Genbank: NW_001471687]. Consequently this information can map both genes. The map locations of other chicken TLRs was determined using cDNA sequences in a BLAT search on the UCSC genome browser [56]. The sequence for chicken TLR2B was shown to contain a gap of unidentified nucleotides. This region was sequenced in both versions of TLR2 and the gap found to be an assembly artefact on both the February 2004 and May 2006 genome builds [Genbank: EF595650].

### Calculation of time of divergence of duplicated genes

Nucleotide alignments of chicken TLR duplications were created using DAMBE [57] which aligns nucleotide sequences to the protein alignment one codon at a time, thus allowing functionally similar areas of each gene to remain aligned and not become victim of codon slippage. Non-synonymous (dN) and synonymous sequence divergence (dS) were estimated with the codeml program in PAML [58] in Runmode = -2 with codonfreq = 2 using the Goldman and Yang [59] substitution model. The substitution rates were estimated using the nucleotide frequencies at each codon position using the F3 × 4 model [58] to estimate the expected codon frequencies.

In order to get an estimate of the age of these duplications (T) the equation, r = K/(2T) [24] was used, where r is the neutral substitution rate per site per year and K is the total synonymous sequence divergence (d_S_) for the total aligned sequence (0.4119 and 0.184 for TLRs 1 like and 2 respectively). We used an overall estimate of r = 1.4 × 10^-9 ^that we obtained by dividing the autosomal sequence divergence between chicken and turkey (0.1008) from Axelsson et al. [60] with the time of divergence between chicken and turkey of 28 × 10^6 ^– 45 × 10^6^[61,62].

## Authors' contributions

Laboratory work, sequence collection and analysis was carried out by NT. BP provided the tissues RT-PCR and carried out linkage mapping on SNP data provided by NT. HMM analysis was performed by DB. SB and NT carried out tests for selection on duplicated chicken genes. This manuscript and figures were prepared by NT with assistance from SB, DG and DB. DB and DG gave joint supervision of this investigation.

## Supplementary Material

Additional file 1 List of accession numbers. Accession numbers of the nucleotide and protein sequences used in this paper. Click here for file

Additional file 2 Structure of known TLR families. Structures linked by { are both equally likely predictions. Click here for file

Additional file 4 Clade containing TLRs 1, 2, 4, 6, 10 and 14 produced by the Neighbour joining method. This figure shows the clade containing TLRs 1, 2, 4, 6, 10 and14, for the full image see Figure 3. Click here for file

Additional file 5 Clade containing TLRs 11, 12, 13, 21 and 22 produced by the Neighbour joining method. This figure shows the clade containing TLRs 11, 12, 13, 21 and 22, for the full image see Figure 3. Click here for file

Additional file 6 Clade containing TLRs 3, 5, 7, 8 and 9 produced by the Neighbour joining method. This figure shows the clade containing TLRs 3, 5, 7, 8 and 9, for the full image see Figure 3. Click here for file

Additional file 7 Clade containing TLRs 1, 2, 4, 6, 10 and 14 produced by the Maximum Parsimony method. This figure shows the clade containing TLRs 1, 2, 4, 6, 10 and 14, for the full image see Figure 4. Click here for file

Additional file 8 Clade containing TLRs 13, 21 and 22 produced by the Maximum Parsimony method. This figure shows the clade containing TLRs 13, 21 and 22, for the full image see Figure 4. Click here for file

Additional file 9 Clade containing TLRs 3, 5, 7, 8, 9, 11 and 12 produced by the Maximum Parsimony method. This figure shows the clade containing TLRs 3, 5, 7, 8, 9, 11 and 12, for the full image see Figure 4. Click here for file

Additional file 3 List of primers used. The PCR and sequencing primers used in this investigation. Click here for file
