# Supplementary material for: Evolution of the chicken Toll-like receptor gene family: A story of gene gain and gene loss
Source: BMC Genomics. 2008 Feb 1;9:62. doi: 10.1186/1471-2164-9-62 (PMC2275738; doi:10.1186/1471-2164-9-62)
Supplement: Additional file 2 — Structure of known TLR families. Structures linked by { are both equally likely predictions. [file 1471-2164-9-62-S2.pdf]

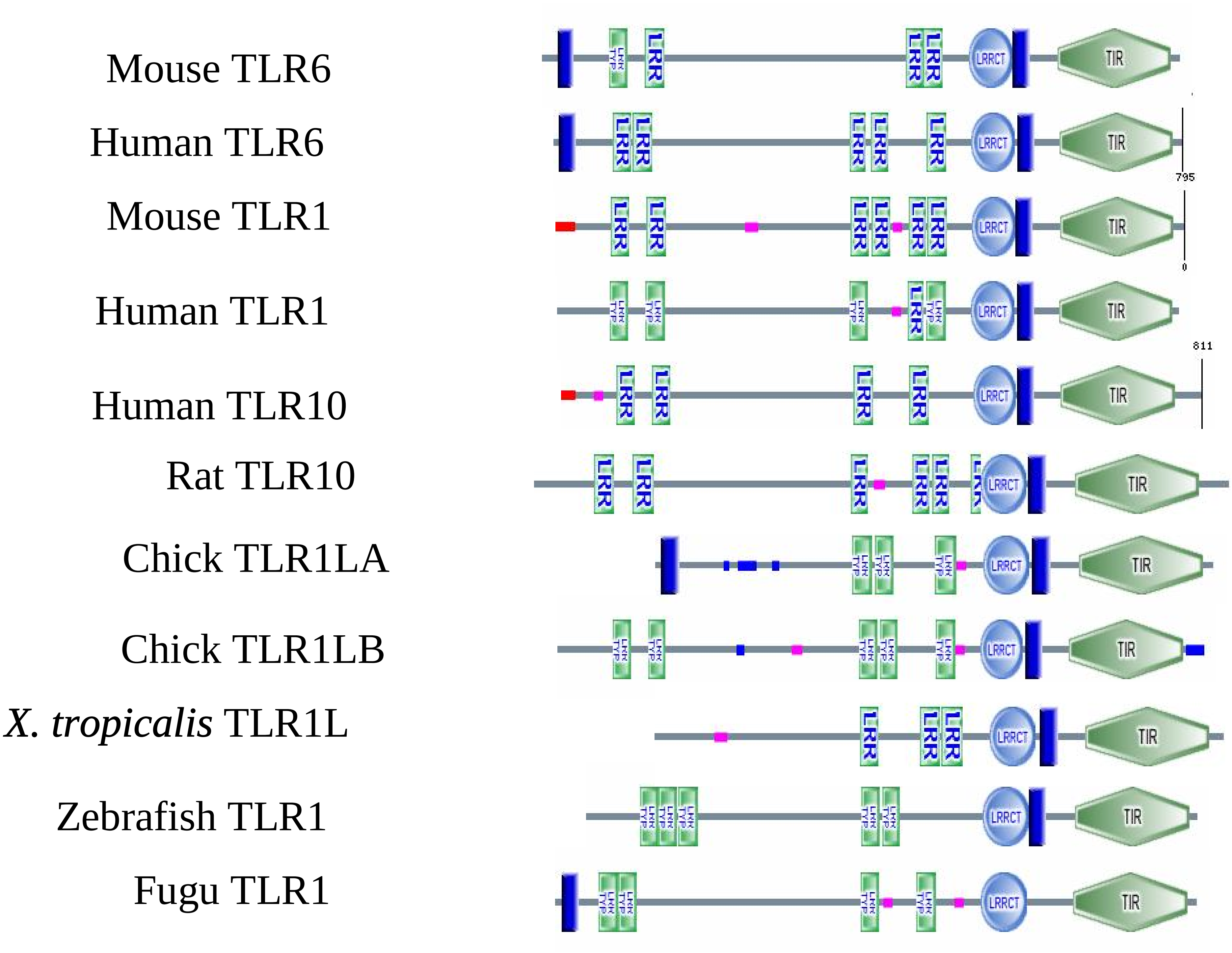

Mouse TLR6
Human TLR6
Mouse TLR1
Human TLR1
Human TLR10
Rat TLR10
Chick TLR1LA
Chick TLR1LB
X. tropicalis TLR1L
Zebrafish TLR1
Fugu TLR1
LRR
LRRCT
TIR
795
811
0

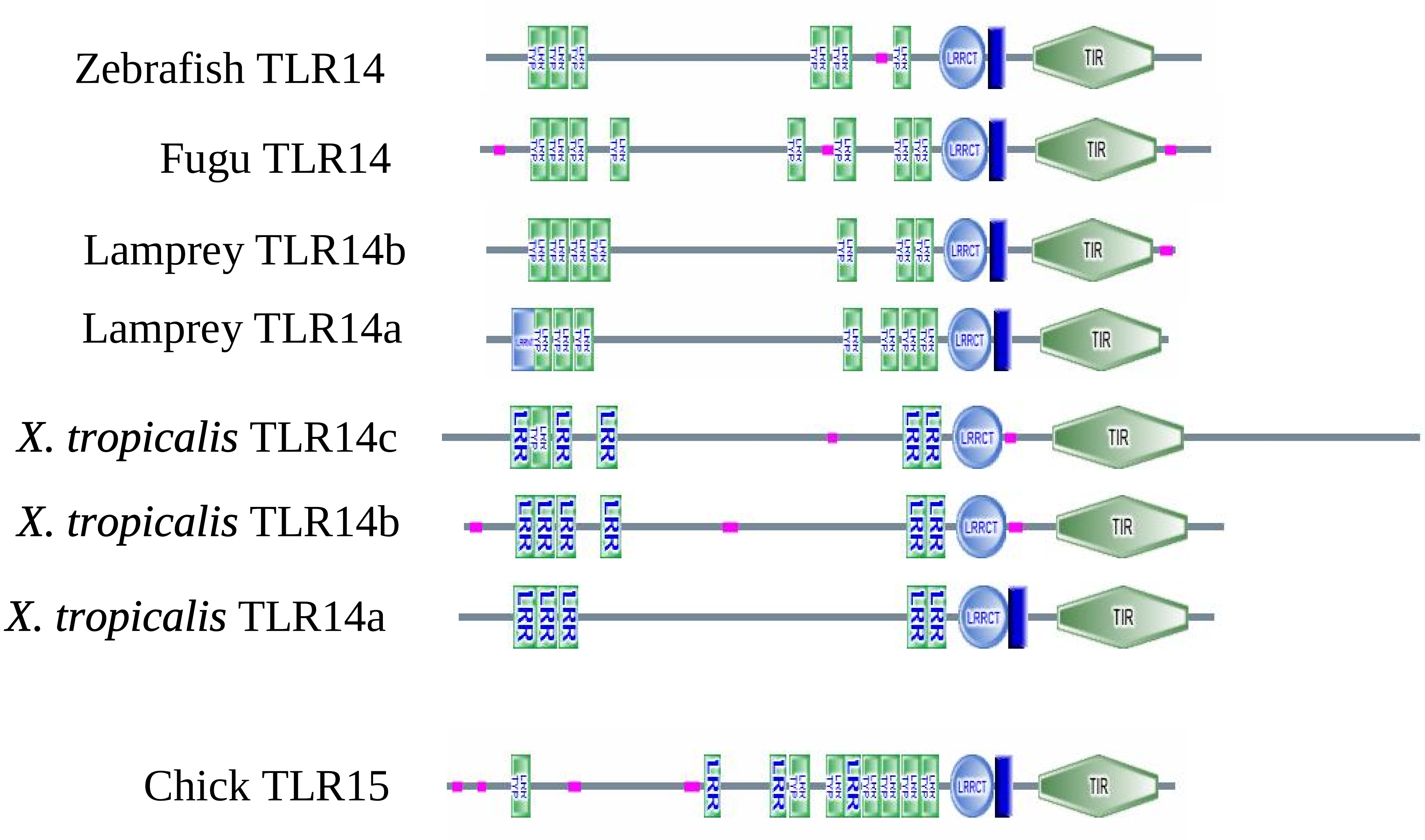
Zebrafish TLR14
Fugu TLR14
Lamprey TLR14b
Lamprey TLR14a
X. tropicalis TLR14c
X. tropicalis TLR14b
X. tropicalis TLR14a
Chick TLR15
LRRCT
TIR
LRR

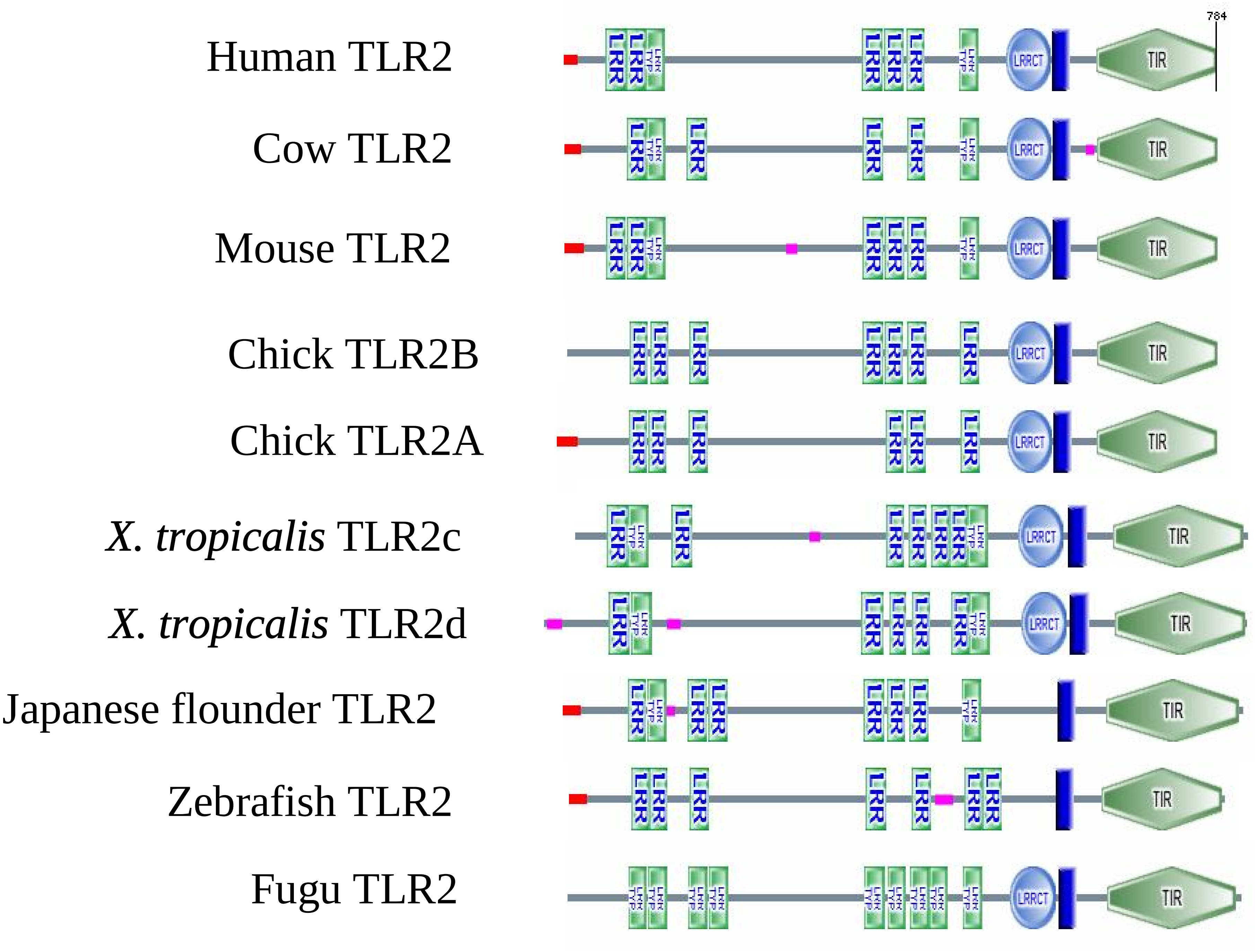

Human TLR2
Cow TLR2
Mouse TLR2
Chick TLR2B
Chick TLR2A
X. tropicalis TLR2c
X. tropicalis TLR2d
Japanese flounder TLR2
Zebrafish TLR2
Fugu TLR2
784
LRR
LRRCT
TIR

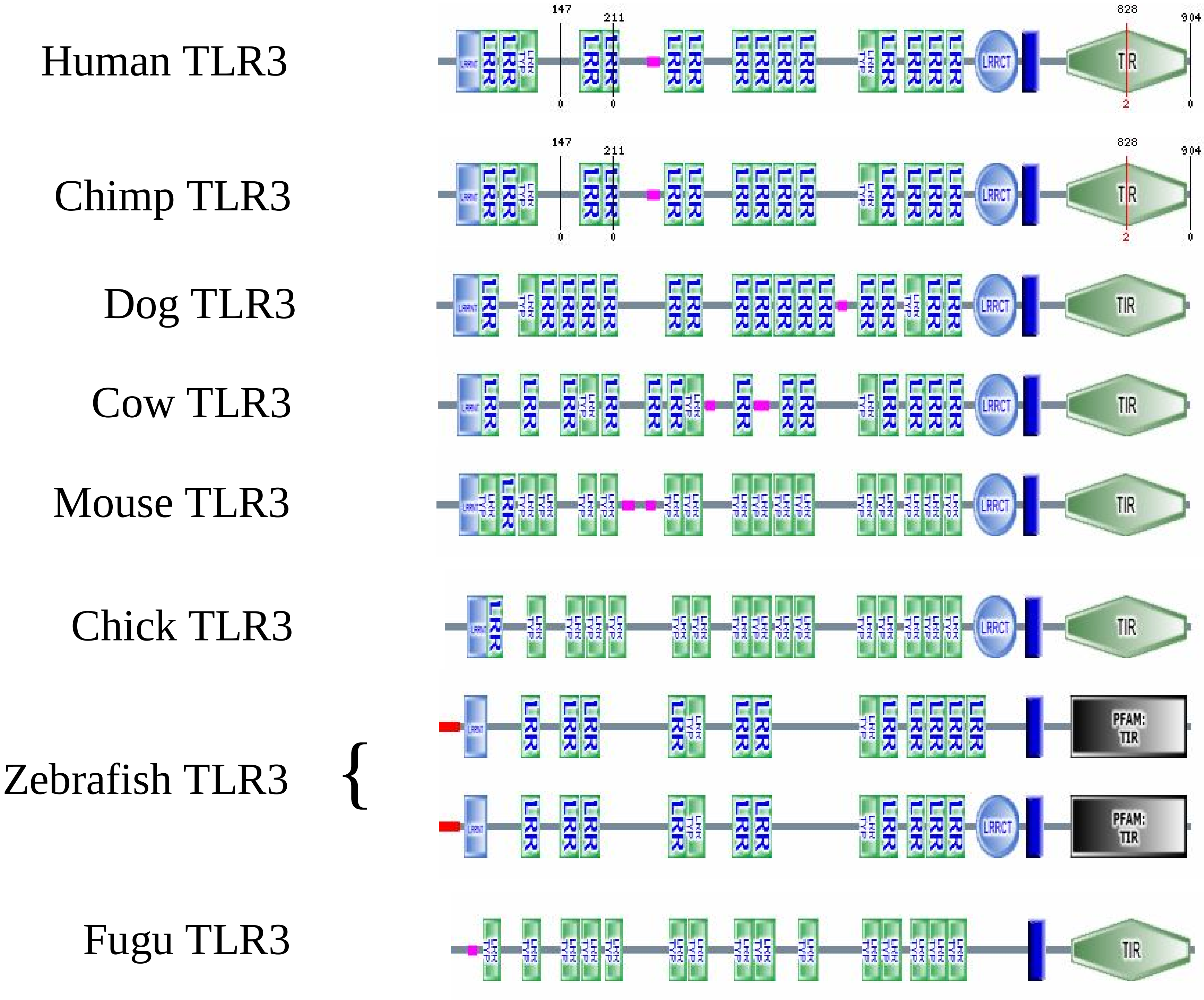

Human TLR3
Chimp TLR3
Dog TLR3
Cow TLR3
Mouse TLR3
Chick TLR3
Zebrafish TLR3
Fugu TLR3
147
211
828
904
LRR
LRRCT
TIR
PFAM: TIR

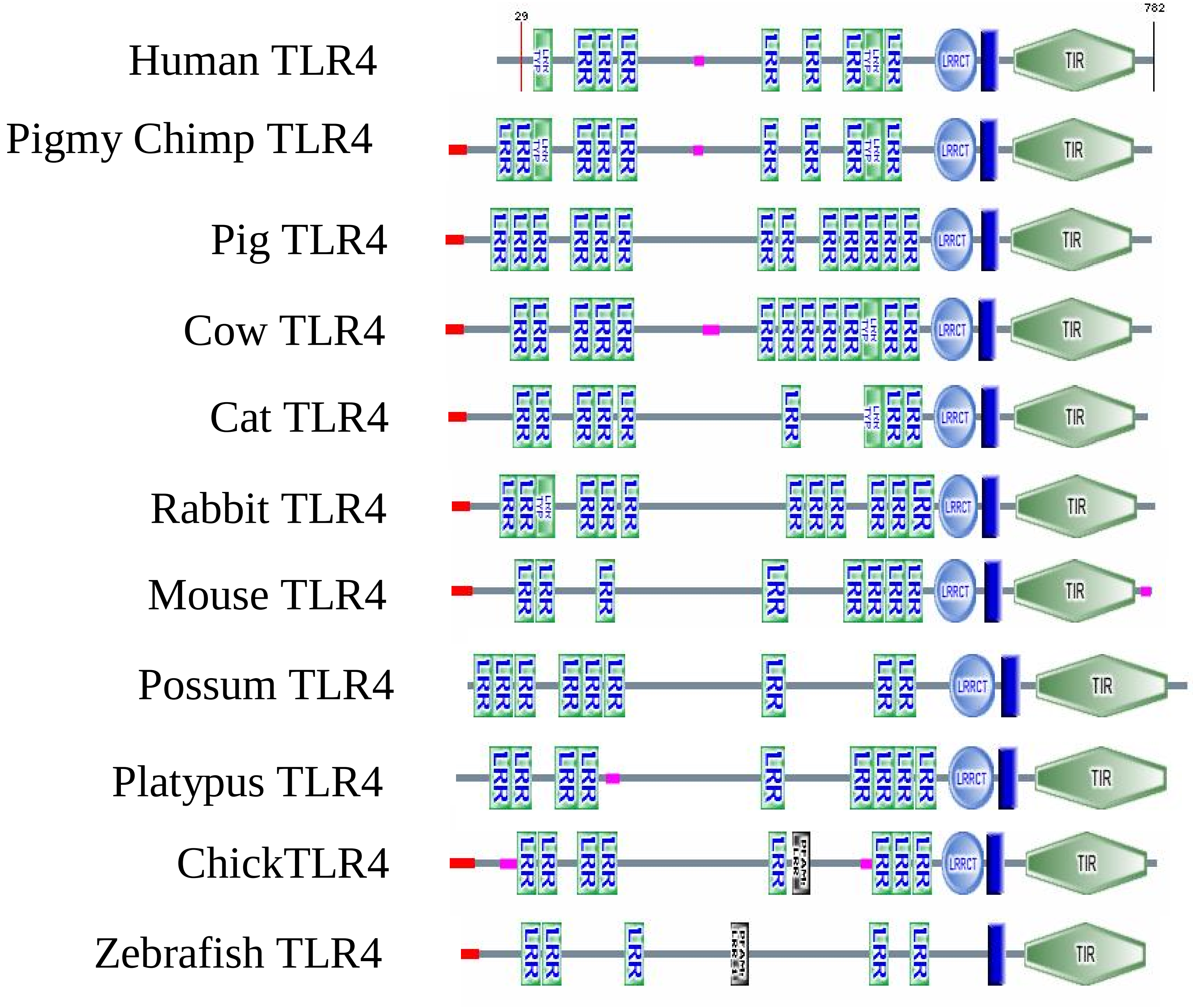
Human TLR4
Pigmy Chimp TLR4
Pig TLR4
Cow TLR4
Cat TLR4
Rabbit TLR4
Mouse TLR4
Possum TLR4
Platypus TLR4
ChickTLR4
Zebrafish TLR4
29
782
LRR
LRRCT
TIR

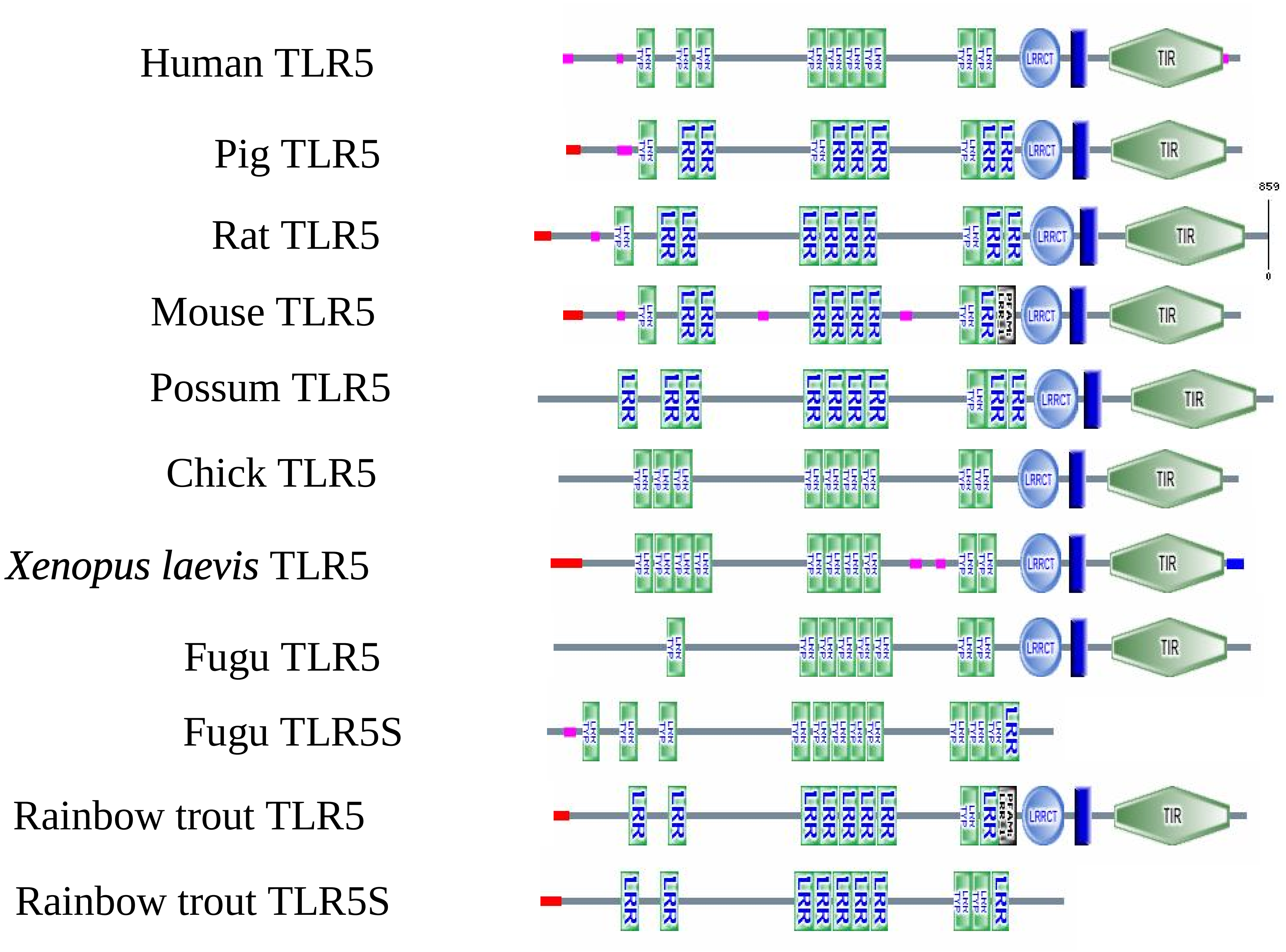

Human TLR5
Pig TLR5
Rat TLR5
Mouse TLR5
Possum TLR5
Chick TLR5
Xenopus laevis TLR5
Fugu TLR5
Fugu TLR5S
Rainbow trout TLR5
Rainbow trout TLR5S
LRR
LRRCT
TIR
859
0

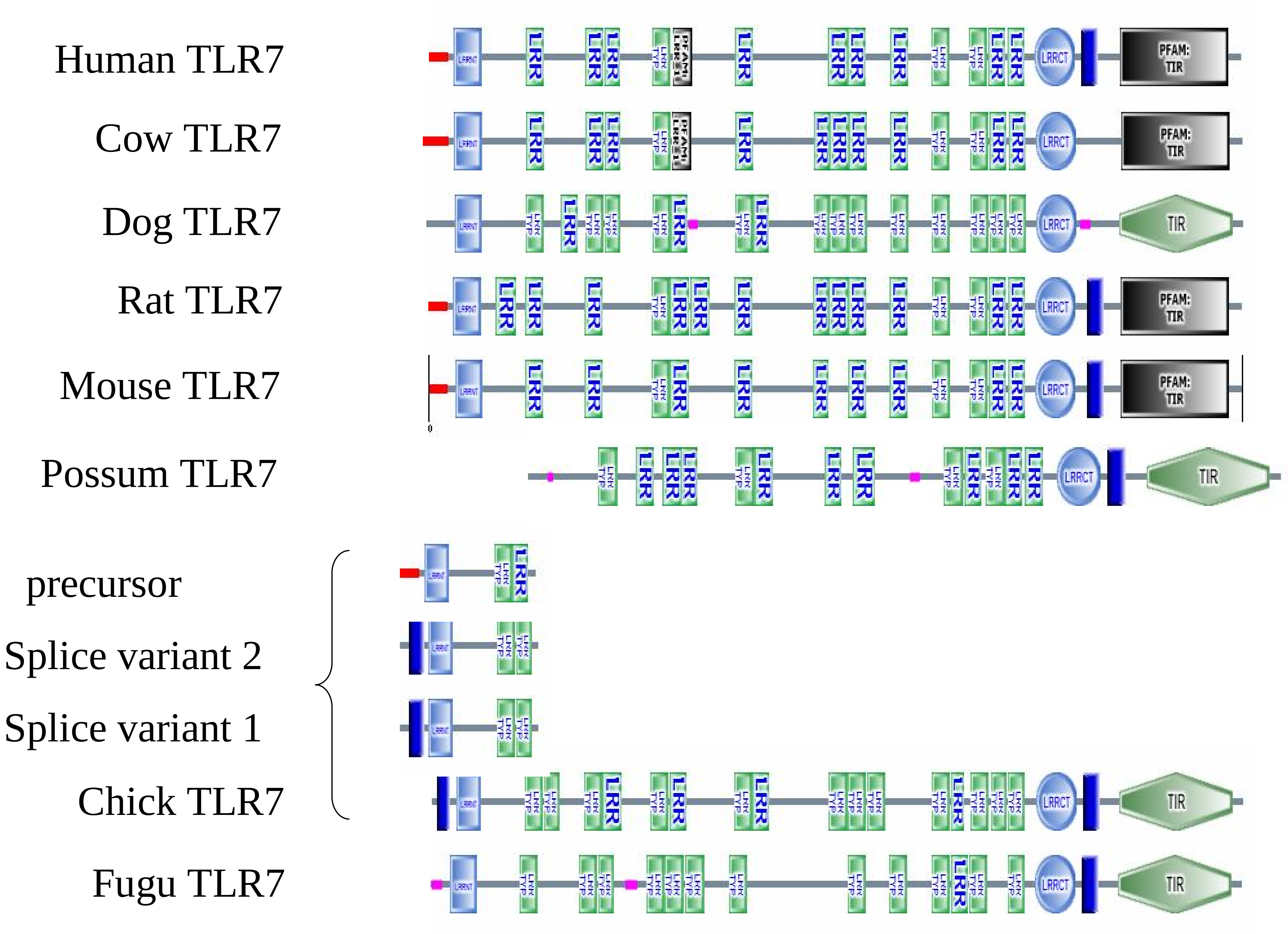
Human TLR7
Cow TLR7
Dog TLR7
Rat TLR7
Mouse TLR7
Possum TLR7
precursor
Splice variant 2
Splice variant 1
Chick TLR7
Fugu TLR7

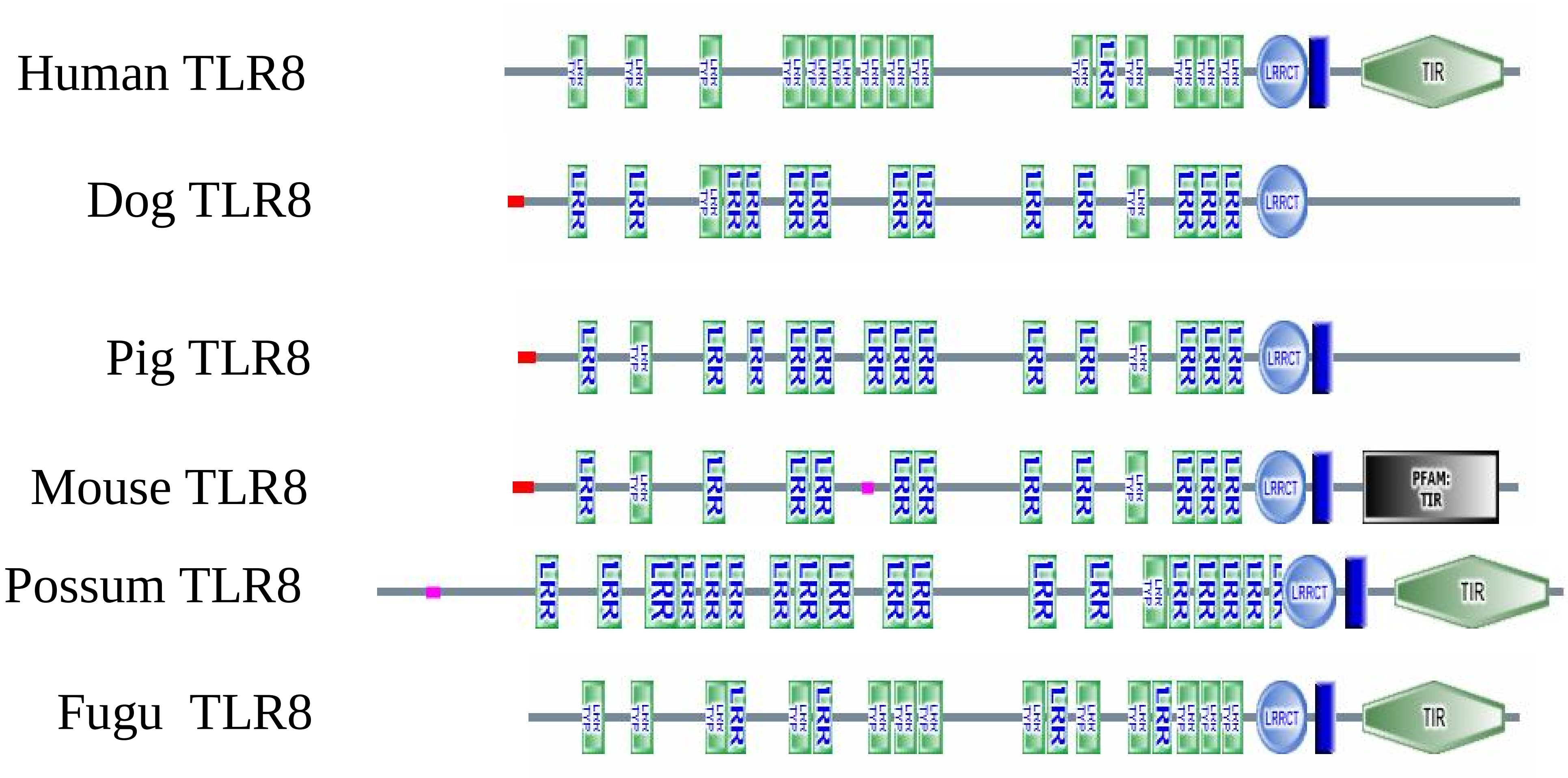
Human TLR8
Dog TLR8
Pig TLR8
Mouse TLR8
Possum TLR8
Fugu TLR8
LRR
LRRCT
TIR
PFAM:
TIR

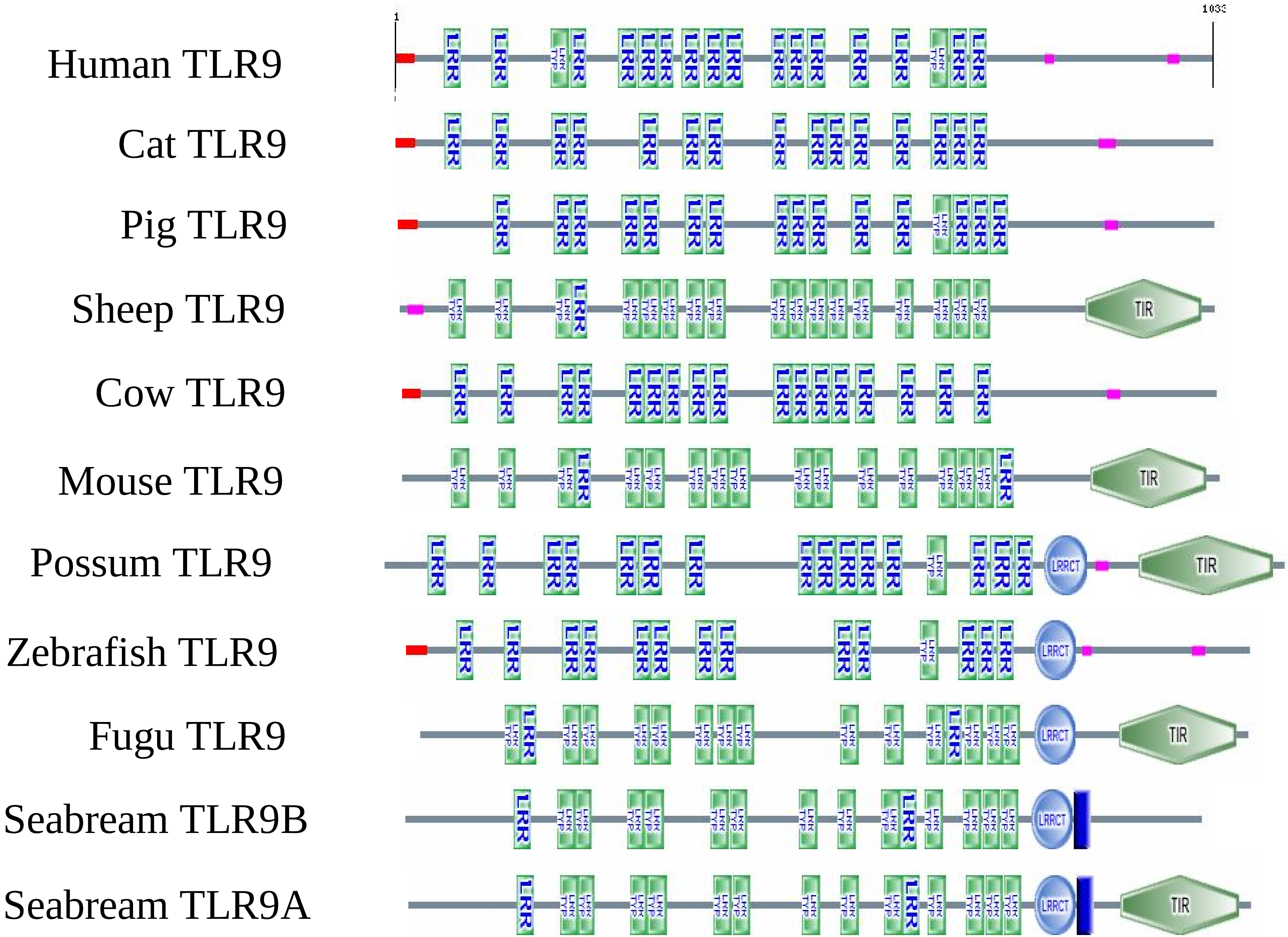
Human TLR9
Cat TLR9
Pig TLR9
Sheep TLR9
Cow TLR9
Mouse TLR9
Possum TLR9
Zebrafish TLR9
Fugu TLR9
Seabream TLR9B
Seabream TLR9A
1
LRR
LRRCT
TIR

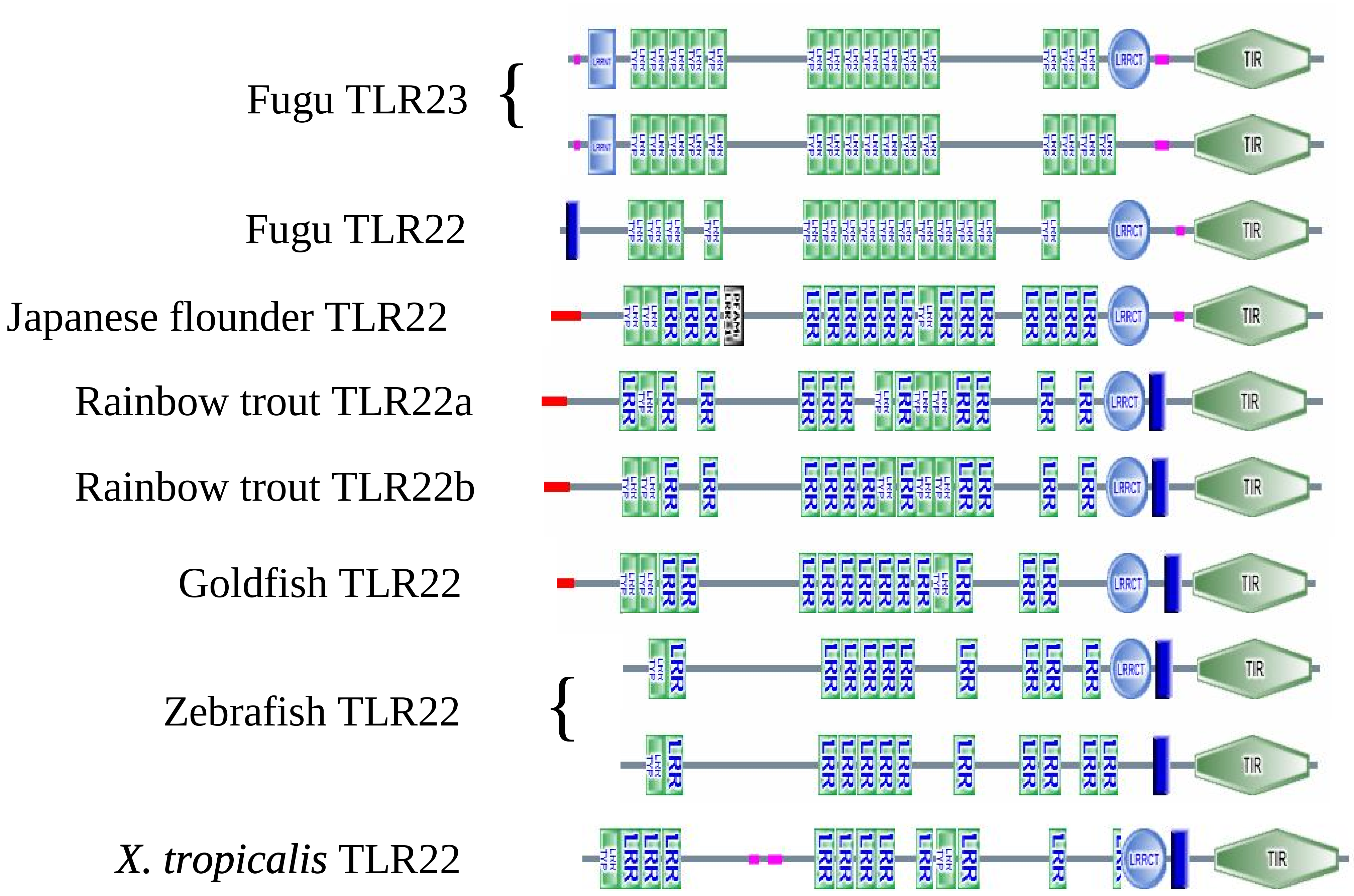

Fugu TLR23
Fugu TLR22
Japanese flounder TLR22
Rainbow trout TLR22a
Rainbow trout TLR22b
Goldfish TLR22
Zebrafish TLR22
X. tropicalis TLR22
LRRNT
LRR
LRRCT
TIR

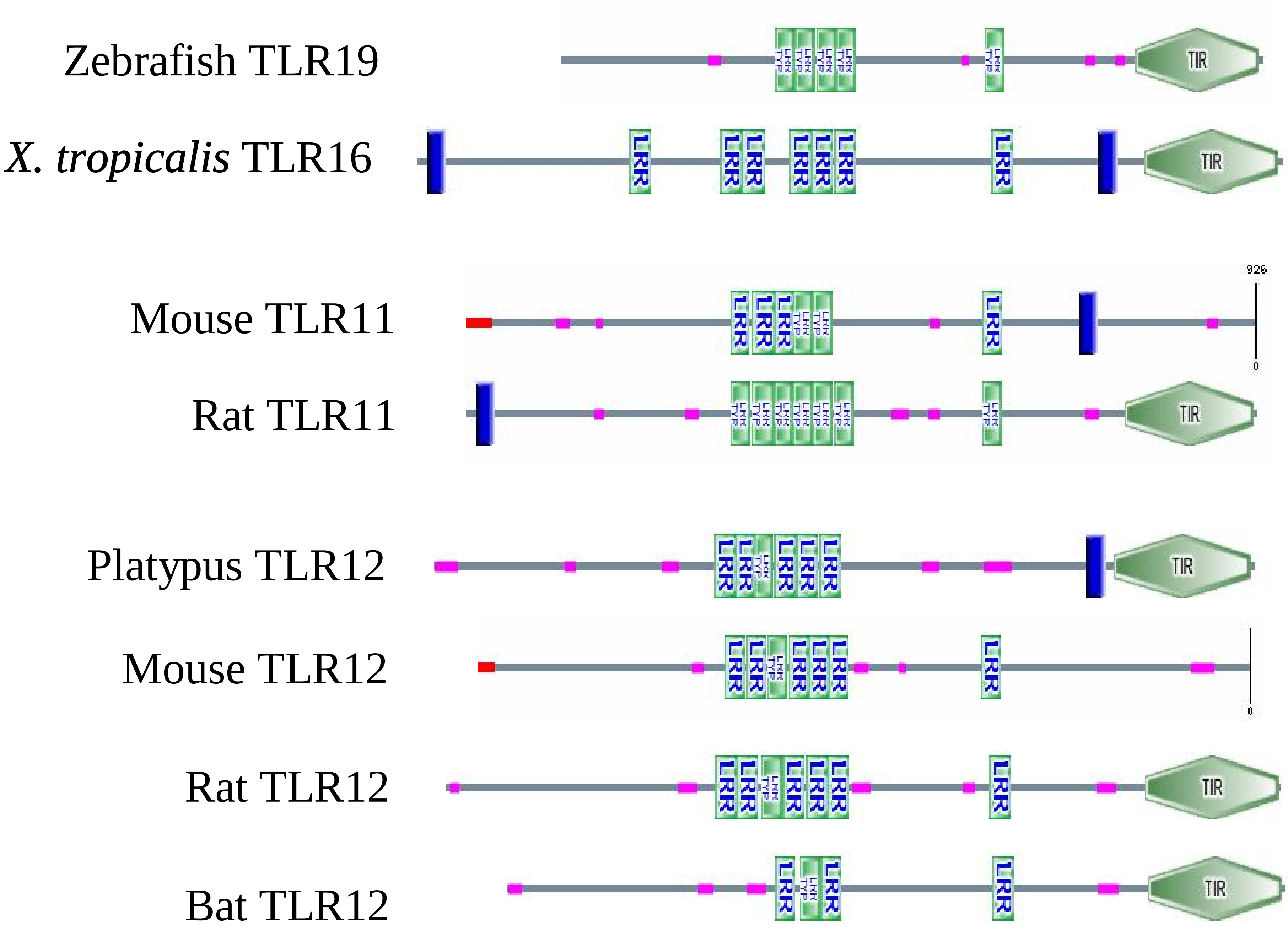

Zebrafish TLR19
X. tropicalis TLR16
Mouse TLR11
Rat TLR11
Platypus TLR12
Mouse TLR12
Rat TLR12
Bat TLR12
LRR
LRR TYP
TIR
926
0

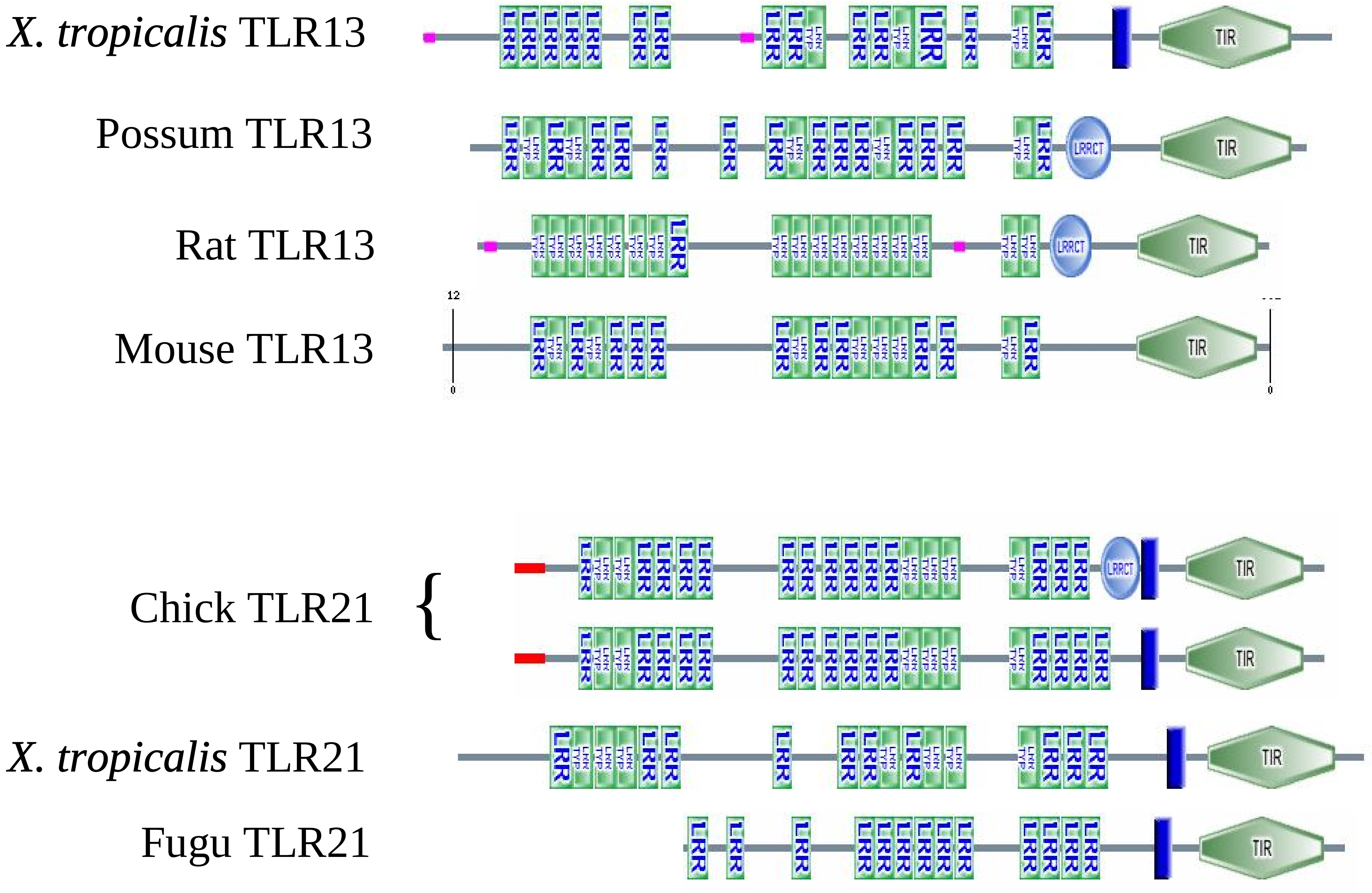

X. tropicalis TLR13
Possum TLR13
Rat TLR13
Mouse TLR13
Chick TLR21
X. tropicalis TLR21
Fugu TLR21
LRR
LRR TYP
LRRCT
TIR
