# Supplementary material for: Evolution of the chicken Toll-like receptor gene family: A story of gene gain and gene loss
Source: BMC Genomics. 2008 Feb 1;9:62. doi: 10.1186/1471-2164-9-62 (PMC2275738; doi:10.1186/1471-2164-9-62)
Supplement: Additional file 4 — Clade containing TLRs 1, 2, 4, 6, 10 and 14 produced by the Neighbour joining method. This figure shows the clade containing TLRs 1, 2, 4, 6, 10 and14, for the full image see Figure 3. [file 1471-2164-9-62-S4.ppt]

## Slide 1
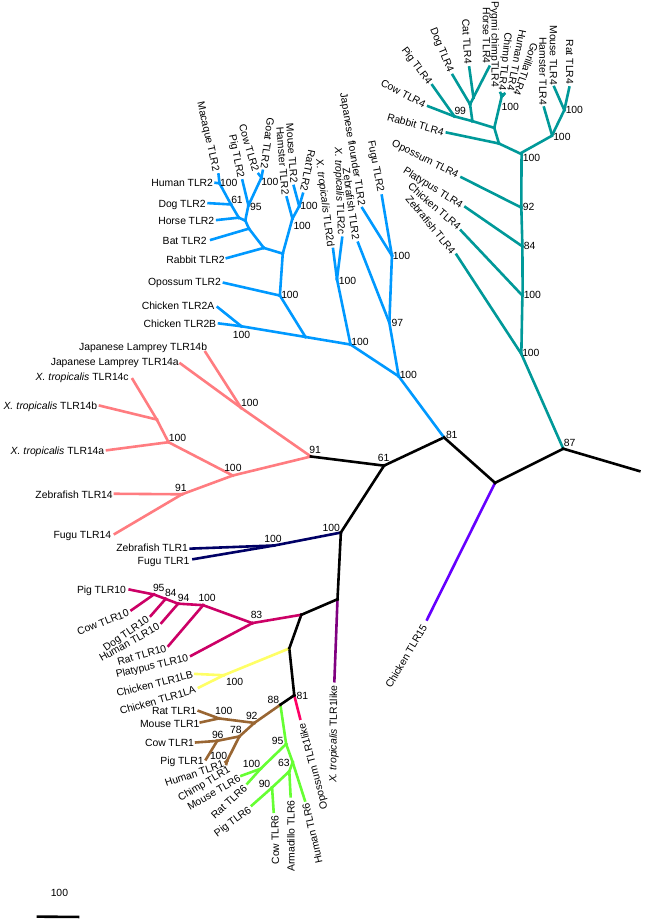

Horse TLR4
Cat TLR4
Pygmi chimpTLR4
Dog TLR4
Mouse TLR4
Human TLR4
Chimp TLR4
Rat TLR4
Pig TLR4
GorillaTLR4
Hamster TLR4
Cow TLR4
100
100
99
Rabbit TLR4
100
Macaque TLR2
Goat TLR2
Cow TLR2
Japanese flounder TLR2
Mouse TLR2
Pig TLR2
100
Opossum TLR4
Hamster TLR2
Fugu TLR2
RatTLR2
100
100
Human TLR2
Platypus TLR4
X. tropicalis TLR2c
61
X. tropicalis TLR2d
Dog TLR2
Zebrafish TLR2
100
Chicken TLR4
95
92
Horse TLR2
Zebrafish TLR4
100
Bat TLR2
84
100
Rabbit TLR2
100
Opossum TLR2
100
100
Chicken TLR2A
97
Chicken TLR2B
100
100
Japanese Lamprey TLR14b
100
Japanese Lamprey TLR14a
100
X. tropicalis TLR14c
100
X. tropicalis TLR14b
81
100
87
91
X. tropicalis TLR14a
61
100
91
Zebrafish TLR14
100
Fugu TLR14
100
Zebrafish TLR1
Fugu TLR1
95
Pig TLR10
84
94
100
83
Cow TLR10
Dog TLR10
Human TLR10
Rat TLR10
Chicken TLR15
Platypus TLR10
100
Chicken TLR1LB
81
88
Chicken TLR1LA
Rat TLR1
100
92
Mouse TLR1
78
X. tropicalis TLR1like
96
95
Cow TLR1
100
Pig TLR1
63
100
Opossum TLR1like
Human TLR1
Chimp TLR1
90
Mouse TLR6
Rat TLR6
Pig TLR6
Human TLR6
Armadillo TLR6
Cow TLR6
100
