# Supplementary material for: Evolution of the chicken Toll-like receptor gene family: A story of gene gain and gene loss
Source: BMC Genomics. 2008 Feb 1;9:62. doi: 10.1186/1471-2164-9-62 (PMC2275738; doi:10.1186/1471-2164-9-62)
Supplement: Additional file 5 — Clade containing TLRs 11, 12, 13, 21 and 22 produced by the Neighbour joining method. This figure shows the clade containing TLRs 11, 12, 13, 21 and 22, for the full image see Figure 3. [file 1471-2164-9-62-S5.ppt]

## Slide 1
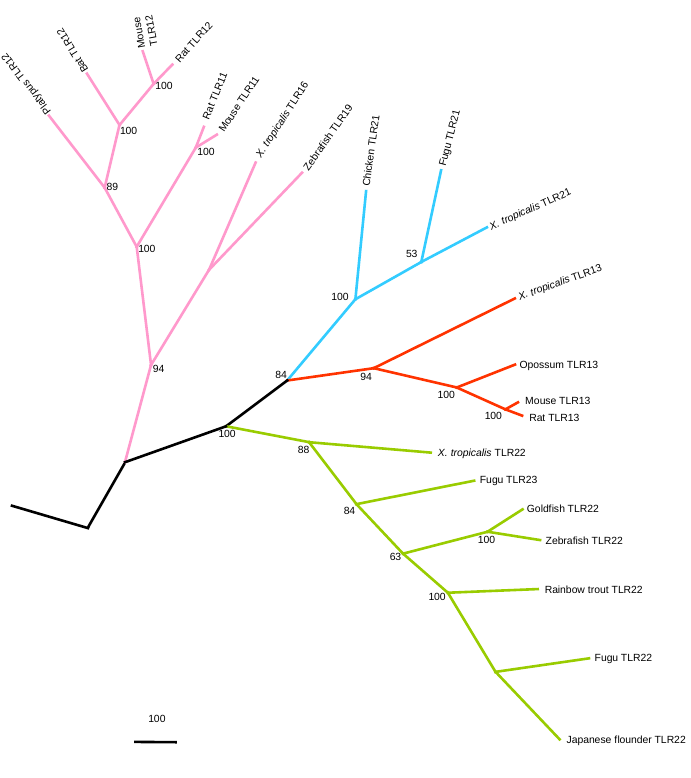

Mouse
TLR12
Rat TLR12
Bat TLR12
Platypus TLR12
100
Rat TLR11
Mouse TLR11
X. tropicalis TLR16
100
Zebrafish TLR19
Fugu TLR21
Chicken TLR21
100
89
X. tropicalis TLR21
100
53
X. tropicalis TLR13
100
Opossum TLR13
94
84
94
100
Mouse TLR13
100
Rat TLR13
100
88
X. tropicalis TLR22
Fugu TLR23
Goldfish TLR22
84
100
Zebrafish TLR22
63
Rainbow trout TLR22
100
Fugu TLR22
100
Japanese flounder TLR22
