# Supplementary material for: Evolution of the chicken Toll-like receptor gene family: A story of gene gain and gene loss
Source: BMC Genomics. 2008 Feb 1;9:62. doi: 10.1186/1471-2164-9-62 (PMC2275738; doi:10.1186/1471-2164-9-62)
Supplement: Additional file 6 — Clade containing TLRs 3, 5, 7, 8 and 9 produced by the Neighbour joining method. This figure shows the clade containing TLRs 3, 5, 7, 8 and 9, for the full image see Figure 3. [file 1471-2164-9-62-S6.ppt]

## Slide 1
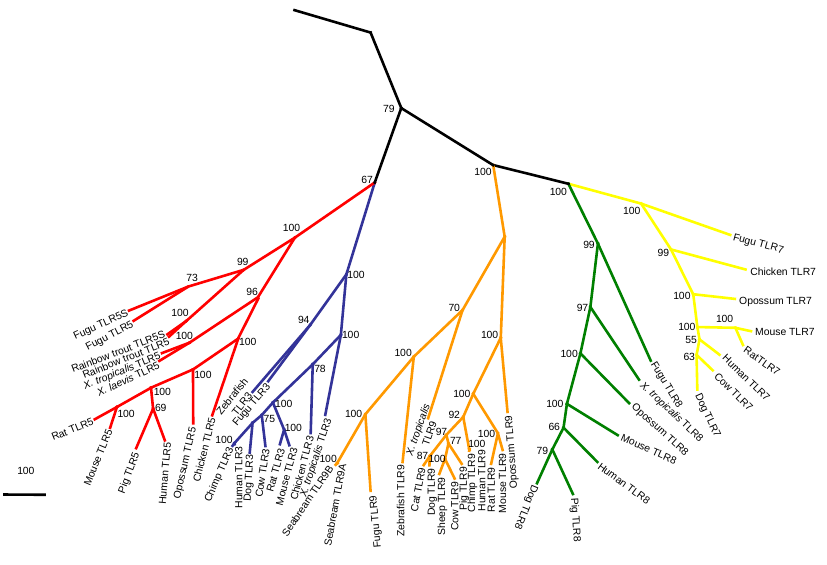

79
100
67
100
100
100
Fugu TLR7
99
99
99
Chicken TLR7
100
73
96
100
Opossum TLR7
70
97
100
100
94
Fugu TLR5S
100
Mouse TLR7
100
Fugu TLR5
100
100
55
100
Rainbow trout TLR5S
100
100
63
Rainbow trout TLR5
RatTLR7
78
X. tropicalis TLR5
100
Human TLR7
X. laevis TLR5
Fugu TLR8
100
Cow TLR7
Zebrafish
TLR3
100
100
100
Fugu TLR3
69
X. tropicalis TLR8
100
100
92
Dog TLR7
75
X. tropicalis
TLR9
66
100
Rat TLR5
Opossum TLR8
97
100
100
77
100
Mouse TLR8
Chicken TLR5
79
Opossum TLR9
87
X. tropicalis TLR3
Mouse TLR5
100
100
Opossum TLR5
Chicken TLR3
Rat TLR3
100
Pig TLR5
Cow TLR3
Human TLR5
Chimp TLR3
Mouse TLR3
Human TLR3
Dog TLR3
Human TLR9
Chimp TLR9
Mouse TLR9
Human TLR8
Pig TLR9
Cat TLR9
Rat TLR9
Dog TLR9
Seabream TLR9B
Zebrafish TLR9
Seabream TLR9A
Cow TLR9
Sheep TLR9
Dog TLR8
Pig TLR8
Fugu TLR9
