# Supplementary material for: Evolution of the chicken Toll-like receptor gene family: A story of gene gain and gene loss
Source: BMC Genomics. 2008 Feb 1;9:62. doi: 10.1186/1471-2164-9-62 (PMC2275738; doi:10.1186/1471-2164-9-62)
Supplement: Additional file 7 — Clade containing TLRs 1, 2, 4, 6, 10 and 14 produced by the Maximum Parsimony method. This figure shows the clade containing TLRs 1, 2, 4, 6, 10 and 14, for the full image see Figure 4. [file 1471-2164-9-62-S7.ppt]

## Slide 1
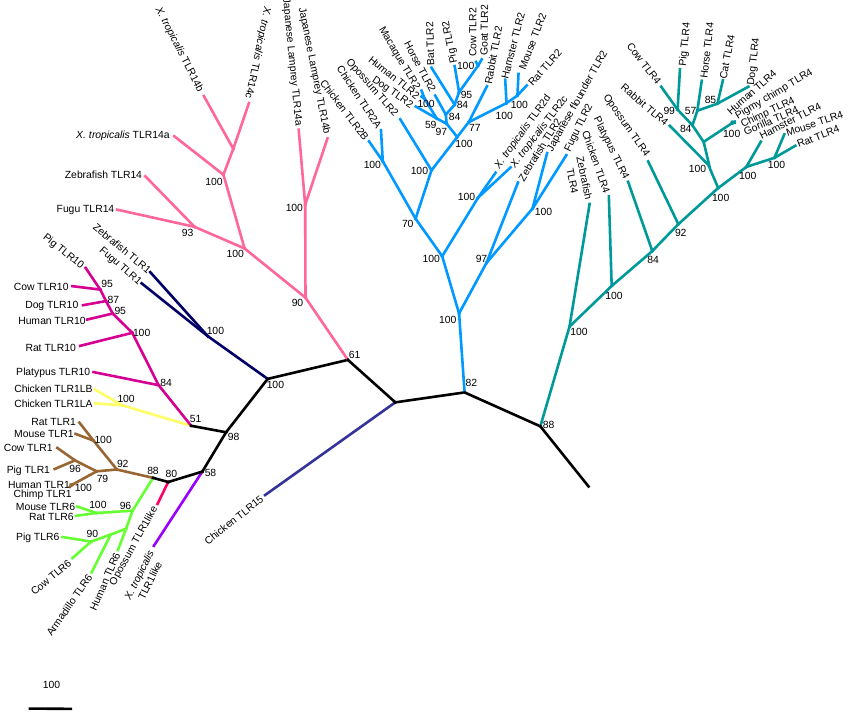

Goat TLR2
Cow TLR2
Mouse TLR2
Pig TLR2
Bat TLR2
Pig TLR4
Hamster TLR2
X. tropicalis TLR14b
Horse TLR4
X. tropicalis TLR14c
Rabbit TLR2
Cat TLR4
Macaque TLR2
Dog TLR4
Japanese Lamprey TLR14a
Cow TLR4
100
Horse TLR2
Rat TLR2
Japanese Lamprey TLR14b
Human TLR2
Opossum TLR2
Human TLR4
Dog TLR2
Pigmy chimp TLR4
95
Chicken TLR2A
85
Japanese flounder TLR2
100
100
Rabbit TLR4
84
57
Chicken TLR2B
99
Chimp TLR4
100
84
Gorilla TLR4
Hamster TLR4
Mouse TLR4
59
Opossum TLR4
Fugu TLR2
77
84
X. tropicalis TLR2c
X. tropicalis TLR2d
97
100
X. tropicalis TLR14a
Rat TLR4
100
Platypus TLR4
Zebrafish TLR2
Chicken TLR4
100
100
100
100
Zebrafish
TLR4
Zebrafish TLR14
100
100
100
100
100
Fugu TLR14
100
70
92
93
Zebrafish TLR1
Pig TLR10
100
100
97
84
Fugu TLR1
95
Cow TLR10
100
87
90
Dog TLR10
95
100
Human TLR10
100
100
100
Rat TLR10
61
Platypus TLR10
84
82
100
Chicken TLR1LB
100
Chicken TLR1LA
51
Rat TLR1
88
Mouse TLR1
98
100
Cow TLR1
92
96
Pig TLR1
88
58
80
79
Human TLR1
100
Chimp TLR1
100
96
Mouse TLR6
Rat TLR6
Chicken TLR15
90
Pig TLR6
Opossum TLR1like
X. tropicalis
TLR1like
Cow TLR6
Human TLR6
Armadillo TLR6
100
