# Supplementary material for: Evolution of the chicken Toll-like receptor gene family: A story of gene gain and gene loss
Source: BMC Genomics. 2008 Feb 1;9:62. doi: 10.1186/1471-2164-9-62 (PMC2275738; doi:10.1186/1471-2164-9-62)
Supplement: Additional file 8 — Clade containing TLRs 13, 21 and 22 produced by the Maximum Parsimony method. This figure shows the clade containing TLRs 13, 21 and 22, for the full image see Figure 4. [file 1471-2164-9-62-S8.ppt]

## Slide 1
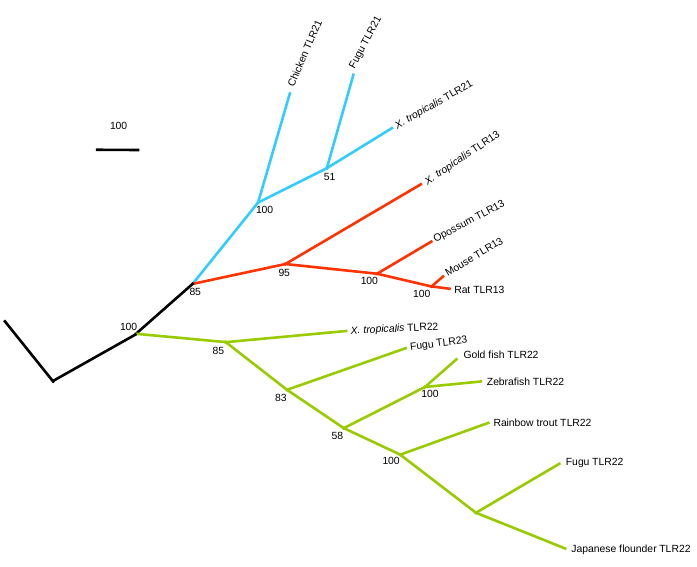

Fugu TLR21
Chicken TLR21
X. tropicalis TLR21
100
X. tropicalis TLR13
51
100
Opossum TLR13
Mouse TLR13
95
100
Rat TLR13
85
100
100
X. tropicalis TLR22
Fugu TLR23
85
Gold fish TLR22
Zebrafish TLR22
100
83
Rainbow trout TLR22
58
100
Fugu TLR22
Japanese flounder TLR22
