# Supplementary material for: Evolution of the chicken Toll-like receptor gene family: A story of gene gain and gene loss
Source: BMC Genomics. 2008 Feb 1;9:62. doi: 10.1186/1471-2164-9-62 (PMC2275738; doi:10.1186/1471-2164-9-62)
Supplement: Additional file 9 — Clade containing TLRs 3, 5, 7, 8, 9, 11 and 12 produced by the Maximum Parsimony method. This figure shows the clade containing TLRs 3, 5, 7, 8, 9, 11 and 12, for the full image see Figure 4. [file 1471-2164-9-62-S9.ppt]

## Slide 1
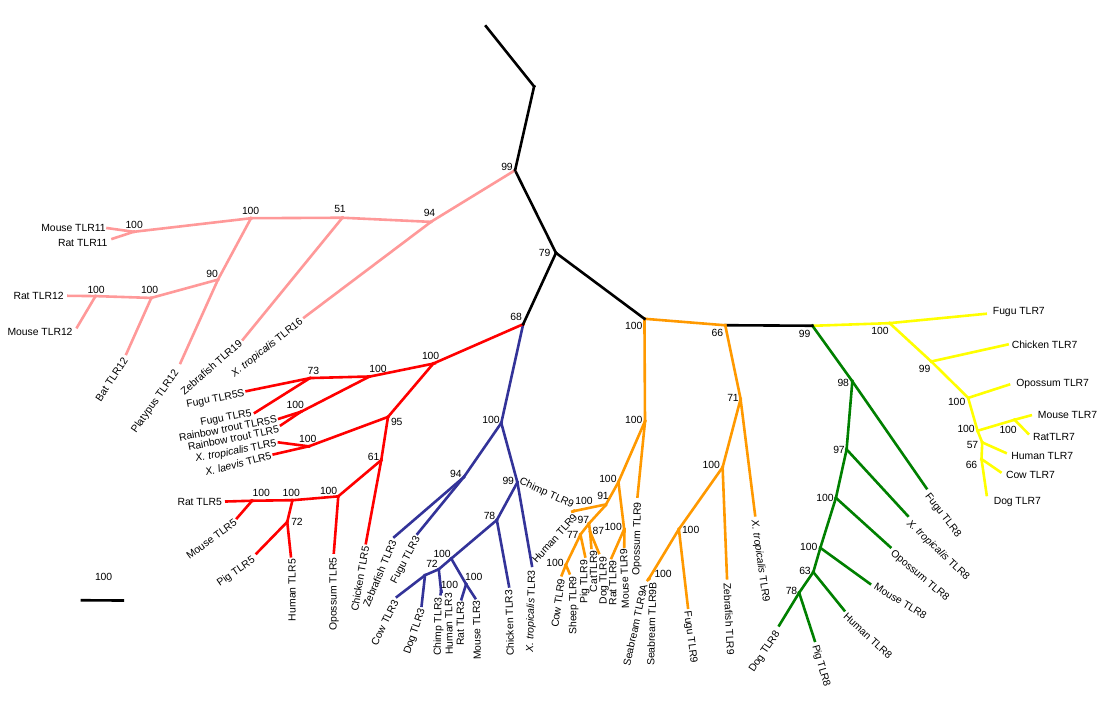

99
51
100
94
100
Mouse TLR11
Rat TLR11
79
90
100
100
Rat TLR12
Fugu TLR7
68
100
100
Mouse TLR12
66
99
Chicken TLR7
X. tropicalis TLR16
100
Zebrafish TLR19
100
99
73
Bat TLR12
98
Opossum TLR7
Fugu TLR5S
71
Platypus TLR12
100
100
Mouse TLR7
Fugu TLR5
100
100
95
Rainbow trout TLR5S
100
100
RatTLR7
Rainbow trout TLR5
100
57
97
X. tropicalis TLR5
Human TLR7
61
X. laevis TLR5
100
66
94
Cow TLR7
100
99
100
Chimp TLR9
100
100
91
100
Dog TLR7
100
Rat TLR5
Fugu TLR8
78
97
72
100
100
87
77
Human TLR9
Mouse TLR5
Opossum TLR9
100
X. tropicalis TLR8
100
Fugu TLR3
X. tropicalis TLR9
100
72
Pig TLR5
63
CatTLR9
100
Zebrafish TLR3
Opossum TLR8
100
100
Chicken TLR5
Mouse TLR9
Dog TLR9
Pig TLR9
Rat TLR9
100
Human TLR5
78
Opossum TLR5
Mouse TLR8
Cow TLR9
Sheep TLR9
X. tropicalis TLR3
Zebrafish TLR9
Cow TLR3
Chicken TLR3
Rat TLR3
Human TLR3
Seabream TLR9B
Seabream TLR9A
Chimp TLR3
Mouse TLR3
Dog TLR3
Human TLR8
Fugu TLR9
Dog TLR8
Pig TLR8
